# Identifying Early Signals From Emerging Public Health Events Using Natural Language Processing

**DOI:** 10.1155/ipid/6176855

**Published:** 2026-03-06

**Authors:** Kelly S. Peterson, Christian Dalton, Andrea Kalvesmaki, JoAnn Vuong, Colton Gordon, Senthil Nachimuthu, Mary Jo Pugh, Makoto M. Jones

**Affiliations:** ^1^ Veterans Health Administration, Office of Analytics and Performance Integration, Washington, District of Columbia, USA; ^2^ Informatics Decision-Enhancement, and Analytic Sciences (IDEAS) Center of Innovation, VA Salt Lake City Health Care System, Salt Lake City, Utah, USA; ^3^ Department of Internal Medicine, Division of Epidemiology, Spencer Fox Eccles School of Medicine University of Utah, Salt Lake City, Utah, USA

## Abstract

Timely detection of emerging public health threats is challenging because the surveillance infrastructure is not yet tuned to the emerging threat. We attempt to identify three nonspecific early signals that might be common across emerging events: public health authority communication, zoonotic exposure mentions, and other pathogen exposure mentions.

**Methods:**

Data from U.S. Department of Veterans Affairs emergency department visits between 2004 and 2024 were used to construct training and validation sets from reportable or emerging infectious diseases identified by historical diagnoses and laboratories. Not all early signal types were extracted using the same method. Rule‐based and transformer models were used in a way to minimize developer and chart reviewer time. We then extracted cases from historic documents among selected diseases.

**Results:**

Positive predictive values for public health authority communication, zoonotic exposure, and other pathogen exposure ranged from 0.615 to 1.0. Target concepts were extracted from over 33 million emergency department visits. Distributions of extracted exposures generally matched expectations for the identified pathogen.

**Conclusion:**

Automated natural language processing methods allow surveillance scaling to large amounts of clinical documents to identify relevant cases. Initial validation compared to manual text review shows that accuracy is acceptable for initial feasibility exploration in biosurveillance efforts.

## 1. Introduction

Rapid identification,​ characterization, and containment of emerging public health events require early detection from available signals. When a novel disease is first observed, it will not have a name or known etiology. When a case definition can be constructed, structured data elements such as ICD‐10 diagnosis codes may have poor PPV and sensitivity [1, 2]. The presence of epidemiologic clues may provide the additional signal necessary to identify an uncommon or unknown infectious disease. These may be documented in the electronic health record (EHR) by astute clinicians as they try to make sense of an unfamiliar disease.

The need to document potential pathogen exposures or communicate with health authorities may provide this extra signal, but this information is not typically captured in structured form, such as by drop‐down list, and is therefore found in free‐text unstructured clinical notes. Since it is not feasible for anything but a very small fraction of notes to be reviewed by analysts, an automated solution would have to scan a very large number of notes and narrow them down for suspicion of an emerging disease to make this possible [3].

Natural language processing (NLP) has been used to extract symptoms from the EHR for syndromic surveillance, potentially identifying symptoms of concerning cases even before confirmation in laboratory results or other diagnostics are available [4–6]. Many NLP targets are nonspecific (e.g., fever) [7, 8] and frequently drawn from chief complaints [9–12] and do little to differentiate routine from concerning infectious diseases. Other efforts have extracted symptoms and personal concerns from social media [13–19], but these too are noisy sources.

Another area of focus has involved NLP to identify epidemiological targets from various sources (e.g., EHR, social media, and questionnaires). One study performed NLP on patient questionnaire responses to identify patterns of zoonotic exposures related to Q fever [20]. Travel has also been highlighted as a critical factor for understanding infectious disease transmission [21–24]. Some prior work has assessed the feasibility of using text extraction methods to identify locations people visit, but the applications were not for public health surveillance since the data sources were social media and narrative texts such as blogs [25–28]. Within clinical surveillance, one study performed NLP on clinical documents to extract mentions of travel history [29].

During our work examining text for signs of emerging diseases, we have noticed two common recurring patterns: documentation of potential infectious exposures and communication with public health authorities [30]. Common exposures include zoonotic or pathogen reservoirs [31, 32], such as mosquitoes [33, 34], or contact with other sick individuals [35]. Patients may affirm or deny exposures, but the presence of such documentation provides insight into the clinician’s differential diagnoses. Meanwhile, public health information sharing may consist of consultation on diagnosis, scheduling laboratory testing by another entity, contact tracing, or reporting on specific conditions.

In this work, we evaluate three automated NLP capabilities to extract (1) communication with public health authorities; (2) zoonotic exposures; and (3) other pathogen exposures.

## 2. Materials and Methods

### 2.1. Ethics Statement

This project was reviewed and approved by the Institutional Review Board at the University of Utah and the VA Salt Lake City Research & Development Committee (IRB#000151154).

### 2.2. Data Source

The data in this study come from the Veterans Health Administration (VHA) Corporate Data Warehouse (CDW) [36]. Patient records for review were selected based on a record of emergency department (ED) encounters between Jan 1, 2004, and May 31, 2024.

To identify potential cases for training, we focused on case definitions of Nationally Notifiable Diseases (NND) enumerated by the CDC National Notifiable Diseases Surveillance System (NNDSS) [37]. For most diseases, inclusion events included ICD‐10‐CM codes or positive laboratories identified by LOINC codes from the Reportable Condition Mapping Table (RCMT) made available via Vocabulary Access and Distribution System (VADS) [38]. For cases of COVID‐19, in addition to positive labs, cases were also included based upon chart review, as many patients had tests performed outside of VHA [39]. Within this set of NND cases, clinical documents for the patient were included if they were entered 24 h before or after their positive lab result, diagnosis code, or chart review.

### 2.3. Preprocessing and Sentence Segmentation

Each of the three capabilities established reference sets that utilize spans of text from within full clinical documents. Each of these was conducted using sentence boundary detection. The solution used for this phase on all capabilities was PyRUSH, which is the default sentence segmentation solution in medspaCy 1.2.0 [40]. The default sentence rules were leveraged such that no rules were added or modified. For two of these capabilities (i.e., Public Health Authority Communication and Other Pathogen Exposures), a single sentence was used as the span of text presented to annotators for review. For zoonotic exposures, a span of 3 sentences was presented, comprised of the sentence containing a potential animal keyword as well as the sentence preceding and following it.

### 2.4. Public Health Authority Communication

To establish a reference set of mentions of public health authority communication, a reviewer annotated clinical documents from our NND document set by marking documents affirmed if they mentioned actual communication (e.g., “*reported details on this case to Ohio Public Health Dept*”), negated if there was an explicit mention that communication had not occurred (e.g., “*these results were not communicated to city infectious disease*”), and not affirmed otherwise (e.g., “*encouraged to follow CDC guidelines*”). Further, if any mention described future or uncertain communication, this was labeled as no communication. For validation, documents were split into 60% training and 40% held‐out validation sets.

**FIGURE 1:**
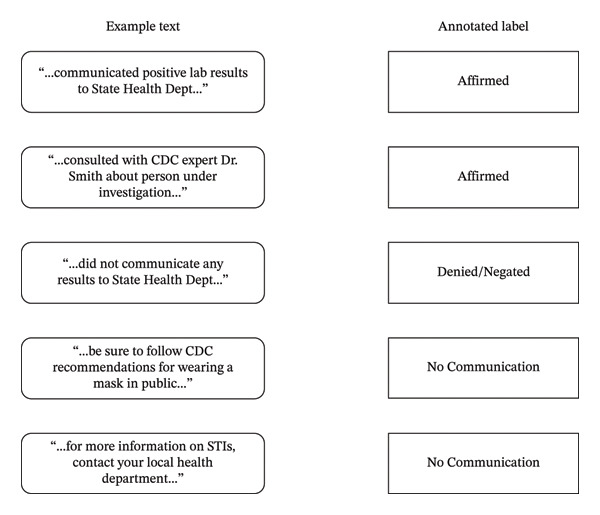
Example text and labels assigned during annotation of public health authority communication.

Some examples of reviewed sentences and labels from this annotation effort are shown in Figure 1.

The NLP task for this capability is that, given a sentence that contains keywords related to potential public health authority, a label is assigned of either Affirmed or otherwise. While annotators did assign labels specifying between Denied/Negated and No Communication, it was decided that for purposes of identifying potentially emerging events, any distinction of communication being denied would have little value. Thus, the NLP task was simplified to a binary classification task on Affirmed status only.

A rule‐based NLP system for identifying public health authority communication was developed using medspaCy [41]. This system leveraged several rules originally developed as part of operational efforts to track COVID‐19 in VA [39]. Rules were added or modified to generalize to other potentially emerging public health events to improve performance on the reference set in this present work. The medspaCy target and context rules for this system have been added in a GitHub repository with a link here to those rules directly^1^. A total of 10 target rules were created for identifying public health authority entities. A total of 12 context rules were created to identify a context category of Affirmed communication. The direction of these context rules being applied was mostly bidirectional, with 10 using this paradigm, with 1 rule specifying forward and 1 backward. The maximum scope for context application was set to either no scope or 8 tokens, with the majority being 7 rules setting this smaller scope of 8 tokens. The resulting system classified a mention of a public health authority as being true for affirmed communication if this context category was applied by the rules and false otherwise.

For comparison, a naïve baseline assumed all keywords were Affirmed. Classification metrics such as PPV, sensitivity, and F1 were calculated for validation of the automated system with respect to the reference set.

### 2.5. Zoonotic Exposures

We used the same NND set to establish a reference set of mentions of potential zoonotic exposures. Documents were processed into text spans if they contained at least one animal keyword. These text spans were comprised of the sentence containing that keyword as well as the sentence before and after. This was decided from an early round in pre‐annotation where the reviewer requested additional context. The vocabulary for keywords was developed from existing literature surveying zoonoses and related vectors [42, 43]. These keywords were also augmented using resources published online [44, 45]. Some of these keywords were specific names for an animal (e.g., giraffe, camel, and mosquito), whereas others were broad terms or categories that are mentioned in literature and online resources for zoonotic transmissions (e.g., livestock, cattle, and insects).

**FIGURE 2:**
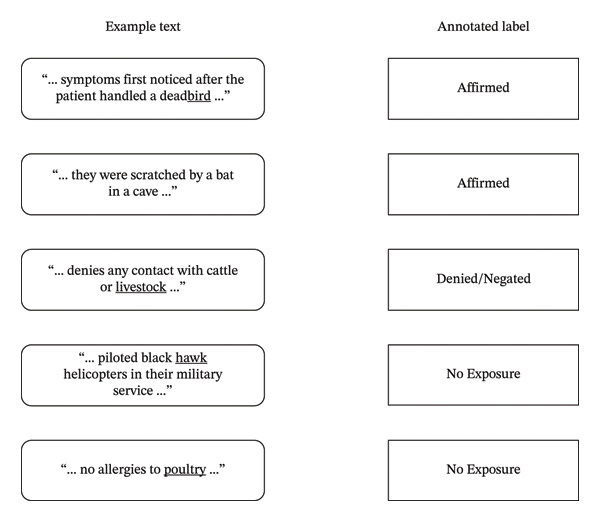
Example text and labels assigned during annotation of animal exposures. While some mentions are Affirmed exposure, others may be Denied or Negated. Other keyword findings were not related to animals or exposures.

Text spans were provided to an annotator, who then assigned each span a category of affirmed, denied/negated, or no exposure to any animal exposure, including pets and farm animals. Some examples of text and associated label annotations are shown in Figure 2.

To efficiently augment the size of our training set since most spans had no exposure, a large language model (LLM) was also used to generate synthetic spans for each category, where the model used was ChatGPT GPT‐4. Several prompts were constructed, such as “*Write 50 more sentences similar to this one: Patient scratched by a monkey on vacation*” or “*Provide example sentences that could be found in a clinical document where the patient was in contact with an animal or insect.”* There were no prompt templates used in this process, and generation parameters such as temperature were kept as defaults. These synthetic examples were added only to the training set such that the validation set was comprised of annotations from actual clinical documents. There was no deduplication or comparison analysis made between the synthetically generated examples and the original training or validation splits. The annotator reviewed these spans using the same criteria. The reference set was split into 60% training and the remaining 40% as a held‐out validation set.

The NLP task for this capability is that, given a sentence containing a keyword potentially related to an animal, a label is assigned of either Affirmed, Denied/Negated, or No Exposure.

A machine learning classification model was trained using the SetFit framework for contrastive learning (i.e., few‐shot learning) [46]. While the SetFit method has been used in other work to evaluate the training effectiveness of fewer examples, all instances in the training set were used at training time. The base LLM used for fine‐tuning was the default sentence transformer model (i.e., paraphrase‐mpnet‐base‐v2) known as Sentence‐BERT [47]. While only this base model was evaluated, other SetFit hyperparameters were optimized by performing a randomized hyperparameter search and 5‐fold cross validation within the training set only. These hyperparameters included the number of epochs, learning rate, and the SetFit parameter of the number of iterations to generate sentence pairs in its contrastive training. Some parameters were left as SetFit defaults, including the batch size of 16 and cosine similarity as the loss function. PPV, Sensitivity, and F1 were calculated for validation of the automated system with respect to the reference set.

After validation, this model was used to infer animal exposure mentions among infectious disease cases which were not part of annotation to examine the distribution of animals mentioned.

### 2.6. Other Pathogen Exposures

We took a similar approach expanding the training set for other pathogen exposures as with animal exposures by annotating clinical documents and augmenting them with synthetic generations from LLMs. These synthetic examples were added only to the training set such that the validation set was comprised of annotations from actual clinical documents. We initially included documents associated with at least one NND. After this, based on a literature review of various pathogens leading to infectious disease exposure, a set of keywords and phrases was generated that would likely be related to exposures without being false positives. For example, the phrase “raw oysters” was added since they may contain *Vibrio* bacteria [48]. Similarly, “contaminated water” was included given that it can lead to human infection by *Leptospira* [49].

**FIGURE 3:**
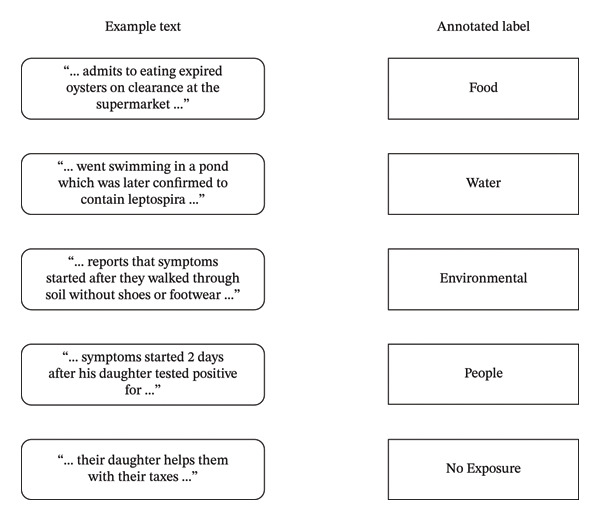
Example text and labels assigned during annotation of other pathogen exposures.

An annotator reviewed the sentences containing these keywords and classified them based on whether the patient was documented as potentially exposed to pathogen exposure categories of Food, Water, People, and Environmental. If the sentence was not a mention of an exposure, it was classified as no exposure. Some examples of text and associated label annotations are shown in Figure 3.

After an annotator reviewed several sentences containing these keywords to classify the exposure status, the next step was to augment synthetic sentences using a generative LLM (ChatGPT as above). Several prompts were constructed, such as “*Write 50 more sentences similar to this one: Patient has experienced significant gastrointestinal distress after consuming raw oysters*” or “*Provide example sentences that could be found in a clinical document where the patient may have contracted a condition due to exposure to food, water, people or the environment*.*”* An annotator then classified these sentences for pathogen exposure categories using the same criteria. The reference set was split into 60% training and the remaining 40% as a held‐out validation set.

The NLP task for this capability is that, given a sentence containing a keyword relating to potential pathogen exposure, a label is assigned of either Affirmed, Denied/Negated, or No Exposure.

The model evaluated for these pathogen exposures used the same methods as the zoonotic exposure model which also used the SetFit framework and the same base LLM (i.e., paraphrase‐mpnet‐base‐v2). While the SetFit method is sometimes used to evaluate the training effectiveness of fewer examples, all instances in the training set were used in training. Like the zoonotic exposure model, while only this single base model was evaluated, other SetFit hyperparameters were optimized by performing a randomized hyperparameter search of 5‐fold cross validation within the training set only. These hyperparameters included the number of epochs, learning rate, and the SetFit parameter of the number of iterations to generate sentence pairs in its contrastive training. Like the zoonotic exposure model, the defaults for batch size and loss function were used. The same classification metrics were also used for validation (i.e., PPV, Sensitivity, and F1).

Following validation, the model was applied to clinical notes associated with infectious disease cases to infer whether there were mentions of pathogens.

## 3. Results

### 3.1. Data

A total of 33,809,595 ED visits were included in this study across 113 distinct VA medical centers. These visits included 5,188,036 unique patients, among whom 87.9% were male, and the average age was 60 years old at the time of their visit. Among self‐identified data in the data warehouse, the distribution of ethnicity across patients was 84.8% Not Hispanic, 7.1% Hispanic, and 8.1% Missing. Distribution of race was 0.7% American Indian or Alaska Native, 20.2% Black or African American, 0.9% Multiracial, 65.9% White, 1.0% Asian, 0.8% Native Hawaiian or Other Pacific Islander, and 10.5% Missing.

### 3.2. Public Health Authority Communication

Among 336,000 documents, a random sample of 288 sentences containing keywords was reviewed to create the reference set. 20.1% of these were reviewed as affirmed.

Within the held‐out validation set, the naïve baseline yielded 20.3% PPV. The classification metrics of this developed rule‐based system and the other two capabilities proposed in this work are shown in Table 1. Confidence intervals were estimated using Wilson score [50].

**TABLE 1:** Summarization of classification metrics for all three automated text pipelines validated on each associated held‐out validation set in this work: communication with public health authorities, zoonotic exposures, and other pathogen exposures.

Text pipeline	PPV (95% CI)	Sensitivity (95% CI)	F1 (95% CI)
Public health authority communication	0.787 (0.595, 0.908)	0.703 (0.515, 0.841)	0.729 (0.593, 0.898)
Zoonotic exposures	0.977 (0.847, 0.995)	0.956 (0.809, 0.984)	0.966 (0.875, 1.0)
Other exposures: environmental	1.0 (0.61, 1.0)	0.286 (0.154, 0.54)	0.444 (0.236, 0.724)
Other exposures: food	1.0 (0.61, 1.0)	0.833 (0.487, 0.974)	0.909 (0.704, 1.0)
Other exposures: people	0.727 (0.524, 0.924)	0.727 (0.524, 0.924)	0.727 (0.585, 0.987)
Other exposures: water	0.615 (0.417, 0.848)	0.800 (0.623, 0.984)	0.696 (0.564, 0.975)
Other exposures: no exposure	0.929 (0.861, 0.965)	0.989 (0.942, 0.998)	0.958 (0.913, 1.0)

Following validation, the developed text pipeline was applied to all documents in the set of NNDs. The prevalence of documented public health authority communication varied by disease category. Some of these diseases and the associated communications are shown in Table 2. Early COVID‐19 cases had the highest prevalence of such communication where over half of early cases had at least one such mention. Meanwhile, other diseases such as dengue and leptospirosis had a relatively lower prevalence.

**TABLE 2:** Percentages of cases with at least one mention of affirmed public health authority communication classified by our proposed model among a subset of NNDs.

Disease category	% of cases with public health authority communication	Total cases
COVID‐19 (early cases)	55.6	27
Dengue	6.1	147
Leptospirosis	0.0	102
mpox	35.7	28
Rabies	17.1	199
Tularemia	15.0	20

**FIGURE 4:**
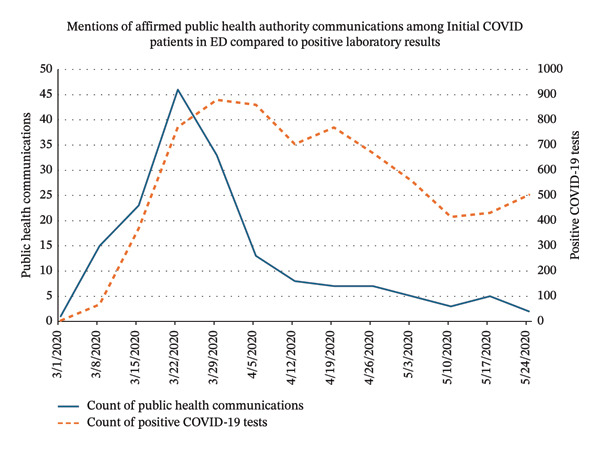
Mentions of affirmed public health authority communication inferred by our proposed model among initial cases of COVID‐19 who also had an ED visit. This series is compared with positive COVID‐19 laboratory events as context for case counts in the healthcare system at that time.

Additionally, this pipeline was applied to documents of COVID‐19 patients who had an encounter in the ED. This time series of communication is compared with positive test results in Figure 4. The documented communications with public health authorities reached its peak in late March 2020 and then decreased over time as the virus is less novel although some communication mentions occurred into April and May of that year.

#### 3.2.1. Zoonotic Exposures

A total of 216 text spans were reviewed for zoonotic exposure. The distribution of labels following annotation is shown in Table 3. Among these, the most prevalent label was No Exposure which comprised approximately half of these annotations.

**TABLE 3:** Distribution of labels among the 216 text spans annotated for zoonotic exposure.

Zoonotic exposure label	Percentage among annotations
Affirmed	40.7
Denied/negated	9.3
No exposure	50.0

Hyperparameter optimization among the training set yielded optimal hyperparameters of 2 epochs, a 0.0002 learning rate, and 8 iterations of sentence pairs. Validation of the zoonotic exposure model was assessed on the held‐out validation set of 87 text spans. The training set was augmented by a total of 51 (39.5%) synthetic examples. The classification metrics of the developed system on this set are shown in Table 1.

After validation, this model was used to perform inferences on a set of 82,937 documents related to several disease categories. Among all documents, the distribution of classifications was 2.7% affirmed, 0.1% denied/negated, and 97.2% no exposure.

**FIGURE 5:**
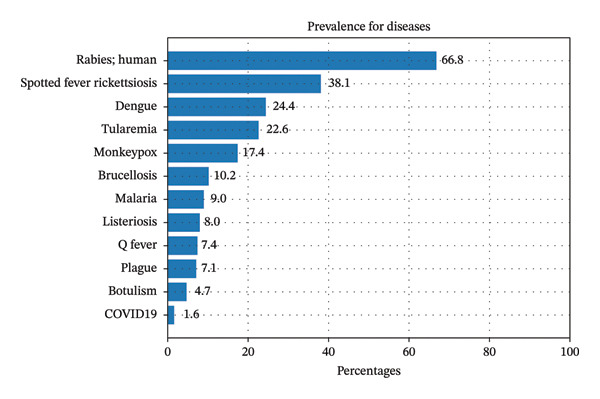
Prevalence of predicted Affirmed Animal Exposures of our proposed model stratified by disease category.

These classifications were analyzed by disease to examine the variance of affirmed exposure. For example, diseases like rabies showed a high prevalence of potential zoonotic exposure mentions, whereas other nonzoonotic diseases such as COVID‐19 were less prevalent. Documents were included in this analysis that had at least one animal keyword so that the model could perform a classification. The distribution of prevalence by disease is shown in Figure 5.

**FIGURE 6:**
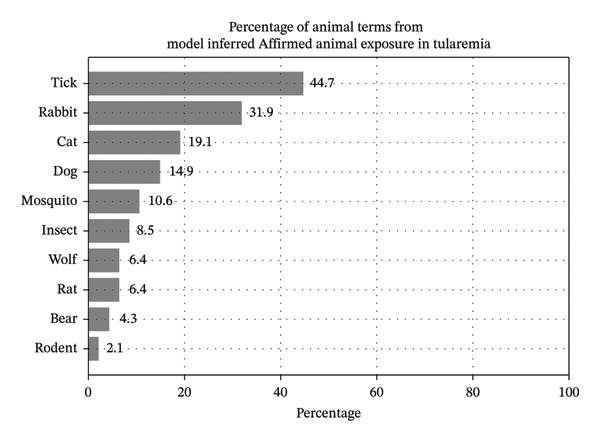
Animal term percentages associated with model inferences of Affirmed exposure among cases of tularemia.

**FIGURE 7:**
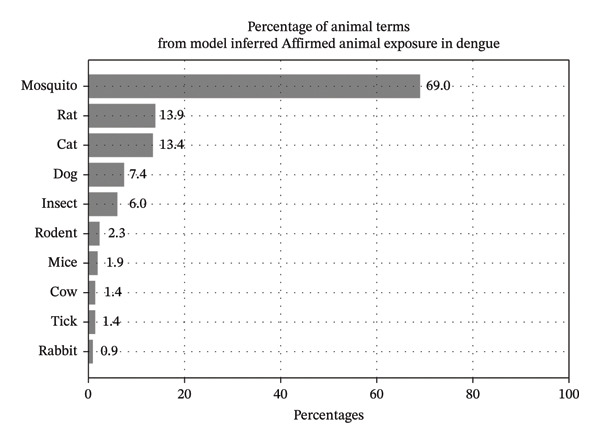
Animal term percentages associated with model inferences of Affirmed exposure among cases of dengue.

Since the proposed model performs its predictions on sentences containing animal keywords, we were able to analyze the most common animal keywords associated with predicted affirmed exposure. The most common keywords are shown by their prevalence percentages for tularemia and dengue in Figures 6 and 7. In these findings, some of the top animal terms for tularemia include “tick” and “rabbit,” whereas dengue includes “mosquito” and “insect.” Note that these mentions in text are simply that there was exposure to the creature and do not necessarily imply that the animal caused the infection. Concretely, since many patients live with pets like cats and dogs, they are technically exposed, although it is unlikely that these diseases were transmitted by their pets.

### 3.3. Other Pathogen Exposures

A total of 377 sentences were reviewed for these other exposures of Food, Water, People and Environmental. The distribution of these labels following annotation is shown in Table 4. Among these, the most prevalent was an annotated label of No Exposure.

**TABLE 4:** Distribution of labels among the 377 sentences annotated for other pathogen exposure.

Other pathogen exposure label	Percentage among annotations
People	8.6
Water	7.9
Environmental	5.4
Food	4.8
No exposure	73.3

Hyperparameter optimization among the training set yielded optimal hyperparameters of 4 epochs, a 0.0005 learning rate, and 4 iterations of sentence pairs. Validation of the model for other pathogens showed that PPV and sensitivity varied by exposure category. The categories with the highest PPV were Food and Environmental, whereas the highest sensitivity among affirmed exposure categories was Food. The training set was augmented by a total of 72 (47.7%) synthetic examples. The validation metrics for this developed system across these exposure categories are shown in Table 1.

After validation, the model was applied to the clinical documents associated with a set of diseases, including early COVID‐19, leptospirosis, and mpox, to analyze the distribution of Affirmed exposure categories according to the trained model for other exposures. This distribution is shown in Table 5. As expected, the disease with the greatest prevalence of inferred exposures to Water and Environmental was leptospirosis since common transmissions for this disease occur in water or soil [51]. Meanwhile, among these cases of COVID‐19 and mpox, the model identified a greater prevalence of mentions of exposure to other people. Nearly half of early COVID‐19 cases and over one‐third of mpox cases mentioned exposures to other people in clinical documentation.

**TABLE 5:** Exposure categories inferred from clinical documents, reported by the percentage of cases that had at least one exposure inferred by our proposed model among a subset of NNDs.

Disease category	% cases with food exposure	% cases with water exposure	% cases with environmental exposure	% cases with people exposure	Total cases
COVID‐19 (early cases)	0.0	0.0	0.0	44.7	38
Leptospirosis	0.5	2.5	2.0	26.0	200
mpox	0.0	0.0	0.0	35.1	37

## 4. Discussion

In this study, we demonstrated the use of automated NLP methods for identifying three distinct concepts related to potential emerging and yet undetected public health events: public health authority communication, zoonotic exposure mentions, and other pathogen exposure mentions. By establishing reference sets reviewed by manual labeling, we developed and trained NLP methods to identify these concepts in unstructured clinical text. These methods were then individually validated by comparison with human judgment in held‐out validation sets.

The accuracy of these automated systems should be sufficient to improve existing biosurveillance of emerging public health events, even if they are novel. Although the sensitivity is relatively lower for some of these systems, their positive predictive value provides an additional signal of a potential emerging disease. As a concrete example, ICD‐10‐CM coding of tularemia may contain false positives from data entry errors, but when combined with documented zoonotic exposures to ticks or rabbits, one may gain greater confidence that the diagnosis may prove correct.

To our knowledge, there has been no previous documented extraction of public health communication from EHR and few instances of animal exposure extractions. We anticipate that the out‐of‐the‐box capabilities of LLMs will likely be applied to many first‐of‐a‐kind extractions from EHRs in the coming years. While we have found few examples related to our work, we still suspect that operational capabilities may have been developed for similar purposes, even if those findings have not been published. Were these efforts to be published, they would be a benefit to the literature.

One limitation of this work is that our approach still depends upon what the clinician chooses to document. If there is no mention in notes of communication with CDC, a patient handling a dead bird, or a patient eating contaminated food, this does not imply that the event has not occurred. While we have observed these concepts being mentioned commonly in several past emerging events such as Zika, COVID‐19, and mpox in VA, there is no guarantee that a clinician will notice or document them.

Additionally, mentions of our targeted concepts do not always imply that the clinician is documenting them as suspicion of an emerging public health event. In some cases, public health authority communication may be simply querying about the availability of laboratory tests. Clinicians may also document that the patient has a pet for emotional support, has an occupation that brings them into contact with animals, or eats certain foods without entertaining an infectious disease.

Another limitation is that while the data for this study comes from a large healthcare system, the population is still majority male and older than in other healthcare systems [52]. Some public health events, whether infectious or otherwise, may present differently in some populations. There could be some events where the population in this study may be relevant and others in which the impact on this population may be lesser. Further, in each annotation task of this study, annotation was performed by one person. As such, inter‐annotator reliability could not be reported in this work, and it would be useful to measure agreement on these annotation efforts in future work.

With respect to the automated NLP systems, several approaches were evaluated. However, the capability of identifying public health authority communication in this study only considered a rules‐based approach. Future work may benefit from evaluating machine learning models. Across all capabilities, several opportunities exist for improving accuracy metrics. Such improvements could come from evaluating other LLMs, which are presently being developed and released at a rapid pace. Additional improvement may also come from additional training data and further hyperparameter optimization at training time. Finally, among the two capabilities that used SetFit for model training, there was no experiment performed to simulate model accuracy with fewer training instances, as all instances were used for maximum performance. Such experiments could be useful in future work to demonstrate how many training examples may be necessary to reach certain levels of classification performance. To support reproducibility of this work, several resources have been made publicly available. These resources include rule resources for the rule‐based identification of public health communications, a small subset of example synthetic data for the exposure capabilities, and code demonstrating how these models are trained and evaluated^2^.

As of the time of this writing, the output of these capabilities is being used to identify potential exposures to highly pathogenic avian influenza (HPAI) or where H5N1 is a potential diagnosis if a patient is documented as having interacted with wild birds, poultry, or livestock. Concern may increase if there exists documentation of a potential exposure to such a pathogen if a patient is documented as consuming raw milk. Even further, a level of concern may be raised if the clinician then communicates findings with the CDC or coordinates with a state health department to test for H5N1. Emerging public health events may take many different shapes and forms, but we will continue to refine and evaluate these systems to prepare to respond to future events.

## 5. Conclusion

In this work, we have proposed methods for automated identification of three distinct early signals of emerging public health events, which have been observed in prior events in the EHRs in our healthcare system. These three signals of public health authority communication, zoonotic exposures, and other pathogen exposures have been validated with respect to human established reference sets. The validity of these systems has been sufficient to begin evaluation for ongoing biosurveillance activities. These will continue to be leveraged in automated systems to prepare for responses to emerging health events. To our knowledge, our work is the first to demonstrate feasibility for identifying these common early signals for biosurveillance purposes. While some prior work has attempted to identify such signals in nonclinical sources, ours is the first to validate such capabilities among millions of patients and documents in a nationwide healthcare system.

## Funding

This work was supported by the U.S. Department of Defense associated with funding opportunity number W81XWH‐21‐PRMRP‐IIRA.

## Disclosure

The funders had no role in study design, data collection and analysis, decision to publish, or preparation of the manuscript. The views expressed in this study are those of the authors and do not necessarily represent the position or policy of the U.S. Department of Veterans Affairs or the U.S. Government.

## Conflicts of Interest

The authors declare no conflicts of interest.

## Endnotes

^1^ https://github.com/burgersmoke/emerging_public_health_nlp/tree/main/resources

^2^ https://github.com/burgersmoke/emerging_public_health_nlp

## Data Availability

Per IRB requirements and VA regulations, patient‐level data from this study cannot be shared directly. Access to source data can be accessed by VA‐credentialed investigators with an approved IRB and proper VA research authorization.
